# 150. Nasal *Staphylococcus aureus* polymerase chain reaction (NSAP): A pediatric antibiotic stewardship tool

**DOI:** 10.1093/ofid/ofad500.223

**Published:** 2023-11-27

**Authors:** Fernandez Marisol, Michael Swindle, Rachel Downey

**Affiliations:** Dell Children's Medical Center of Central Texas, Dell Medical School and UT Austin, Austin, Texas; Dell Medical School at the University of Texas ay Austin; Dell Children's Medical Center of Central Texas, Austin, Texas; Dell Children's Medical Center of Central Texas, Austin, Texas

## Abstract

**Background:**

In recent years, literature has flourished analyzing nasal *Staphylococcus aureus* polymerase chain reaction (NSAP) diagnostic performance in patients with pneumonia. Fewer data are available for other diagnoses or in pediatrics.

This study aims to determine the baseline use of NSAP results in discontinuation of anti- methicillin resistant *S. aureus* (MRSA) coverage in pediatric patients with bone/joint infection, complicated pneumonia, and skin and soft tissue infection (SSTI). The second aim is to calculate sensitivity and specificity of this test to help determine its utility as a tool to de-escalate antibiotic coverage.

**Figure 1.**
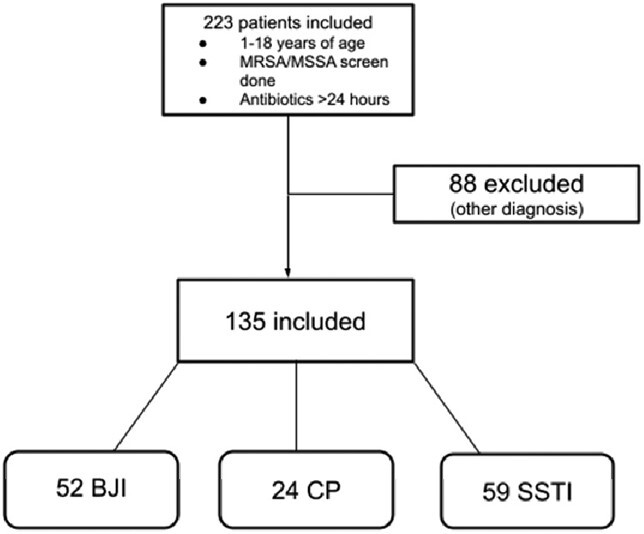
Inclusion and exclusion

**Figure 2.**
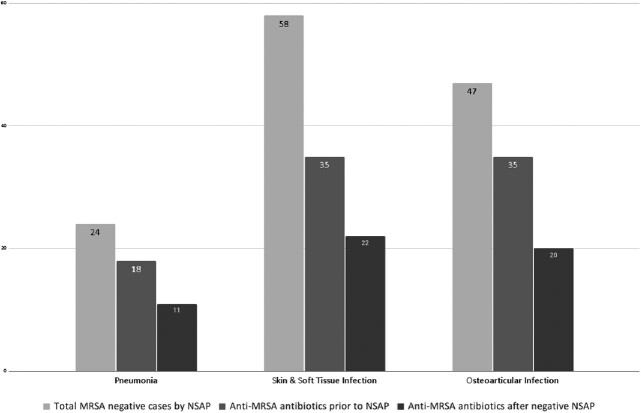
Anti-MRSA antibiotic use before and after negative NSAP testing

**Methods:**

This is a single center, retrospective chart review study of patients with NSAP performed from March 2016 to September 2021. Figure 1 describes case inclusion and exclusion. Empiric antibiotic data were collected and cases were categorized according to antibiotic use with or without MRSA coverage before and directly after NSAP testing. Changes to antibiotics with regard to MRSA coverage were assumed to be related to NSAP results. NSAP results were compared to blood and other site cultures and molecular testing (if done) to determine sensitivity and specificity.

**Results:**

Among the 135 cases included in the analysis, NSAP testing was negative for MRSA in 129 cases. Figure 2 describes MRSA coverage before and after negative NSAP results. False negative rate was < 1% (1/129).

Six cases were positive for MRSA by NSAP (4%). Empiric antibiotics for 4/6 cases provided MRSA coverage. Following positive NSAP results, MRSA coverage was initiated in 1 additional case. Cultures were congruently positive in 4/6 (67%) of these cases.

**Conclusion:**

Institutional use of NSAP testing can result in a decrease of unnecessary anti-MRSA antibiotics. NSAP testing allowed for de-escalation of antibiotics in 40% of cases with negative results. In this sample, the false negative rate was low at < 1%, which, along with similar studies, could give precedence for a system-wide approach for deescalating antibiotics using NSAP. MRSA NSAP screening had sensitivity of 80% and specificity of 98% when compared to conventional tests.

**Disclosures:**

**All Authors**: No reported disclosures

